# Direct conversion of human fibroblasts into hepatocyte-like cells by ATF5, PROX1, FOXA2, FOXA3, and HNF4A transduction

**DOI:** 10.1038/s41598-017-16856-7

**Published:** 2017-11-30

**Authors:** Daiki Nakamori, Hiroki Akamine, Kazuo Takayama, Fuminori Sakurai, Hiroyuki Mizuguchi

**Affiliations:** 1 Laboratory of Biochemistry and Molecular Biology, Graduate School of Pharmaceutical Sciences, Osaka University, Osaka, 565-0871 Japan; 2 PRESTO, Japan Science and Technology Agency, Saitama, 332-0012 Japan; 3 Laboratory of Hepatocyte Regulation, National Institute of Biomedical Innovation, Health and Nutrition, Osaka, 567-0085 Japan; 4 Laboratory of Regulatory Sciences for Oligonucleotide Therapeutics, Clinical Drug Development Project, Graduate School of Pharmaceutical Sciences, Osaka University, Osaka, 565-0871 Japan; 5 Global Center for Medical Engineering and Informatics, Osaka University, Osaka, 565-0871 Japan

## Abstract

Recently, it has been reported that human hepatocyte-like cells can be generated from fibroblasts by direct reprogramming technology. However, the conversion efficiency of human induced hepatocyte-like cells (hiHeps) is not high enough. In addition, comparative analysis with the existing models of hepatocytes, such as human iPS cell-derived hepatocyte-like cells and primary human hepatocytes, has not been sufficiently carried out. In this study, we screened hepatic transcription factors for efficient direct hepatic reprogramming and compared hepatic functions between hiHeps and other existing hepatocyte models. We found that human fibroblasts were efficiently converted into hiHeps by using a combination of ATF5, PROX1, FOXA2, FOXA3, and HNF4A (albumin+/alpha-1 antitrypsin+ cells = 27%, asialoglycoprotein receptor 1+ cells = 22%). The *CYP* expression levels and CYP activities in hiHeps were higher than those in human iPS cell-derived hepatocyte-like cells, but lower than those in short-term (4 hr) cultured primary human hepatocytes and primary human hepatocytes collected immediately after thawing. These results suggested that functional hiHeps could be efficiently generated by ATF5, PROX1, FOXA2, FOXA3, and HNF4A transduction. We believe that hiHeps generated by our method will be useful for the drug-discovery activities such as hepatotoxicity screening and drug metabolism tests.

## Introduction

Hepatocyte-like cells differentiated from human iPS cells (iPS-Hepa) are expected to be applied for liver transplantation, drug metabolism tests, and hepatotoxicity screening. Human iPS cells can be generated from somatic cells such as fibroblasts and peripheral blood mononuclear cells by the transduction of Yamanaka factors (OCT3/4, SOX2, KLF4, and c-Myc)^1,2^. However, it takes a long time to establish human iPS cells and also to differentiate hepatocyte-like cells. In addition, human iPS-Hepa have the risk of generating teratomas due to the contamination of residual undifferentiated iPS cells when they are applied for transplantation. Direct reprogramming technology has the potential to solve these problems. Recently, several studies reported methods for the direct conversion of fibroblasts into hepatocyte-like cells without establishing iPS cells^3–11^. However, each of these methods uses a different combination of hepatic transcription factors for the direct reprogramming as described below. Huang *et al*. have shown that a lentiviral vector-mediated transduction of FOXA3, HNF1A, and HNF4A could transdifferentiate human embryonic fibroblasts into human induced hepatocytes (hiHeps) with purity of 20%^7^. Lim *et al*. have also shown that HNF1A transduction alone is sufficient for direct hepatic reprogramming in the presence of small molecules^10^. In this study, we decided to perform a screening for efficient direct hepatic reprogramming by using the hepatic transcription factors employed in these previous studies. Because our ultimate goal is to apply hiHeps for drug-discovery research, we attempted to establish an efficient method for human iHeps, rather than mouse iHeps.

In this decade, the differentiation technology of iPS-Hepa has been greatly improved. Indeed, many studies have reported the generation of almost homogeneous human iPS-Hepa^12–17^. However, the activities of drug metabolizing enzymes, such as cytochrome P450 (CYP), in human iPS-Hepa are still lower than those in primary human hepatocytes (PHH)^14,18,19^. In addition, it has been reported that human iPS-Hepa retain some of the properties of fetal hepatocytes. On the other hand, hiHeps are not well characterized. A comparative analysis of hepatic functions between hiHeps and existing hepatocyte models (human iPS-Hepa and PHH) would thus be indispensable to assess the value of hiHeps in drug-discovery study.

In this study, we screened 9 hepatic transcription factors to establish an optimal method for efficient direct hepatic reprogramming. To investigate whether hiHeps have the potential to be utilized in drug-discovery studies, the expression of hepatic markers and hepatic functions of hiHeps were compared with those of existing hepatocyte models (human iPS-Hepa and PHH).

## Results

### Screening of hepatic transcription factors

In this study, we attempted to generate hiHeps from human fetal fibroblasts, MRC5 cells (Fig. S1A). First, the efficiency of LV vector mediated-transduction into MRC5 cells was confirmed by using a Venus (modified green fluorescent protein)-expressing LV vector (LV-Venus) (Fig. S1B). The percentage of Venus-positive cells was measured at day 3 after the LV-Venus transduction. Almost homogenous transduction could be performed by using 1,000 vector particle (VP)/cell of LV vectors.

**Figure 1:**
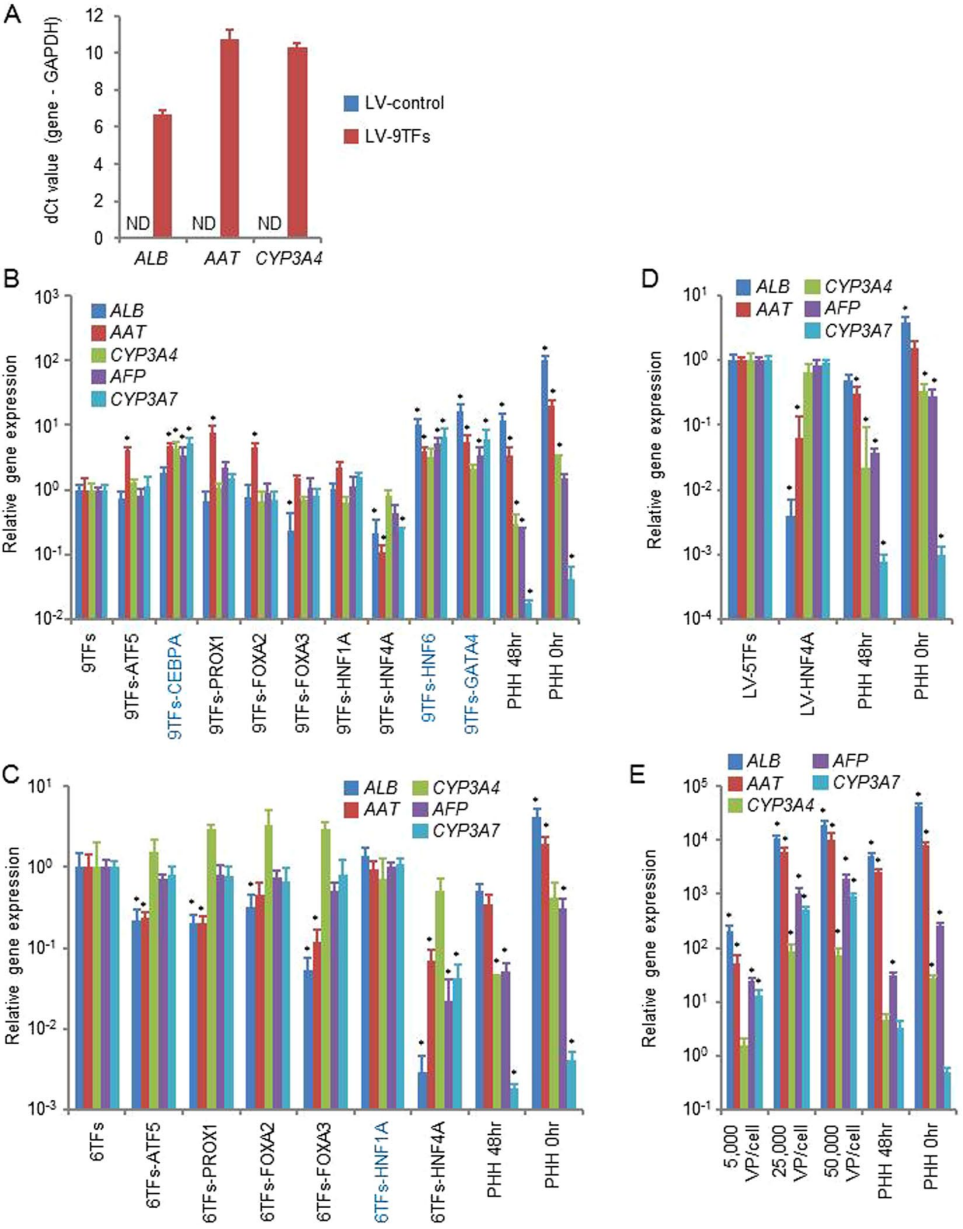
Generation of human induced hepatocyte-like cells (hiHeps) from human fetal fibroblasts. (**A**) Human fetal fibroblasts (MRC-5 cells) were transduced with 5,000 VP/cell/each vector of nine transcription factors (9TFs)-expressing LV vectors (LV-9TFs) for 12 hr, and cultured until day 28. The hepatic gene (*ALB*, *AAT*, and *CYP3A4*) expression levels were measured by real-time RT-PCR. ND: Not detected. (**B**) MRC-5 cells were transduced with LV-9TFs or LV-8TFs (9TFs-ATF5, 9TFs-CEBPA, 9TFs-PROX1, 9TFs-FOXA2, 9TFs-FOXA3, 9TFs-HNF1A, 9TFs-HNF4A, 9TFs-HNF6, or 9TFs-GATA4) for 12 hr, and cultured until day 28. In the case of combination transduction of multiple LV vectors, 5,000 VP/cell of each LV-TF were transduced. The hepatic gene (*ALB*, *AAT*, and *CYP3A4*) and fetal-specific hepatic gene (*AFP* and *CYP3A7*) expression levels were then measured by real-time RT-PCR. The gene expression levels in LV-9TF-transduced MRC5 cells were taken as 1.0. *p < 0.05; **p < 0.01 (vs LV-9TFs). (**C**) MRC5 cells were transduced with LV-6TFs or LV-5TFs (6TFs-ATF5, 6TFs-PROX1, 6TFs-FOXA2, 6TFs-FOXA3, 6TFs-HNF1A, or 6TFs-HNF4A) for 12 hr, and cultured until day 28. In the case of combination transduction of multiple LV vectors, 5,000 VP/cell of each LV-TF were transduced. The hepatic gene (*ALB*, *AAT*, and *CYP3A4*) and fetal-specific hepatic gene (*AFP* and *CYP3A7*) expression levels were then measured by real-time RT-PCR. The gene expression levels in LV-6TF-transduced MRC5 cells were taken as 1.0. *p < 0.05; **p < 0.01 (vs LV-6TFs). (**D**) MRC5 cells were transduced with LV-5TFs or LV-HNF4A for 12 hr, and cultured until day 28. In the case of combination transduction of multiple LV vectors, 5,000 VP/cell of each LV-TF were transduced. The hepatic gene (*ALB*, *AAT*, and *CYP3A4*) and fetal-specific hepatic gene (*AFP* and *CYP3A7*) expression levels were then measured by real-time RT-PCR. The gene expression levels in LV-5TF-transduced MRC5 cells were taken as 1.0. *p < 0.05; **p < 0.01 (vs LV-5TFs). (**E**) MRC5 cells were transduced with 5,000, 25,000, or 50,000 VP/cell of each LV-TF for 12 hr, and cultured until day 28. The hepatic gene (*ALB*, *AAT*, and *CYP3A4*) and fetal-specific hepatic gene (*AFP* and *CYP3A7*) expression levels were then measured by real-time RT-PCR. The gene expression levels in LV-5TF-transduced MRC5 cells were taken as 1.0. *p < 0.05; **p < 0.01 (vs 500 VP/cell). All data are represented as means ± SD (*n* = 3). PHH 48 hr: PHH cultured for 48 hr after plating; PHH 0 hr: PHH collected immediately after thawing.

To perform a hepatic transcription factor screening for efficient direct hepatic reprogramming, we used LV vectors expressing the hepatic transcription factors ATF5, CEBPA, PROX1, FOXA2, FOXA3, HNF1A, HNF4A, HNF6, and GATA4 (9TFs). First, all of these LV vectors (LV-9TFs) were transduced into MRC5 cells at 5,000 VP/cell/each vector, and the transduced cells were cultured in a hepatocyte culture medium (HCM). At day 28, the gene expression levels of the hepatic markers, *albumin* (*ALB)*, *α-1 antitrypsin* (*AAT*), and *CYP3A4*, in the LV-9TF-transduced cells were greatly increased as compared with those in the LV-control-transduced cells (Fig. 1A). Next, to determine which of the 9 candidates were critical, we examined the effect of withdrawal of individual hepatic transcription factors from a pool of transduced candidate genes on the generation of hiHeps (Fig. 1B). The gene expression levels of hepatic markers (*ALB*, *AAT*, and *CYP3A4*) and fetal-specific hepatic markers (*alpha-fetoprotein (AFP)* and *CYP3A7*) were elevated by withdrawal of CEBPA, HNF6, and GATA4, suggesting that these three hepatic transcription factors may suppress direct hepatic reprogramming. In addition, to determine which of the 6 candidates (ATF5, PROX1, FOXA2, FOXA3, HNF1A, and HNF4A (6TFs)) were critical, we examined the effect of withdrawal of individual hepatic transcription factors from the pool of transduced candidate genes on the generation of hiHeps (Fig. 1C). The gene expression levels of *ALB*, *AAT*, *CYP3A4*, *AFP*, and *CYP3A7* were not changed by the withdrawal of HNF1A, suggesting that HNF1A might not play an important role in direct hepatic reprogramming. We also confirmed that HNF4A is the most important hepatic transcription factor for the generation of hiHeps, because the gene expression levels of *ALB*, *AAT*, *CYP3A4*, *AFP*, and *CYP3A7* were markedly decreased by the withdrawal of HNF4A (Fig. 1B,C). Interestingly, hiHeps could be generated by transducing only HNF4A (Figs 1D, S2), although the *ALB* and *AAT* expression levels (Fig. 1D), ALB secretion capacity (Fig. S2A), and percentage of ASGR1-positive cells (Fig. S2C) in the HNF4A-transduced hiHeps were lower than those in the LV-5TF (ATF5, PROX1, FOXA2, FOXA3, and HNF4A)-transduced-hiHeps. Taken together, these results suggest that hiHeps could be efficiently generated by using the following combination of 5TFs: ATF5, PROX1, FOXA2, FOXA3, and HNF4A. However, the expression ratios of ALB/AFP and CYP3A4/CYP3A7 in hiHeps were significantly lower than that in PHH, but higher than that in iPS-Hepa (Fig. S3). This result suggests that hiHeps retain a fetal phenotype as compared with PHH. We also investigated the optimal amount of the LV vectors (Fig. 1E). The expression levels of *ALB*, *AAT*, *CYP3A4*, *AFP*, and *CYP3A7* reached almost plateau levels by using 25,000 VP/cell/each vector. In the following experiments, the MRC5 cells were transduced with 25,000 VP/cell of each LV vector.

### Sequential gene expression analysis during the direct reprogramming process

**Figure 2:**
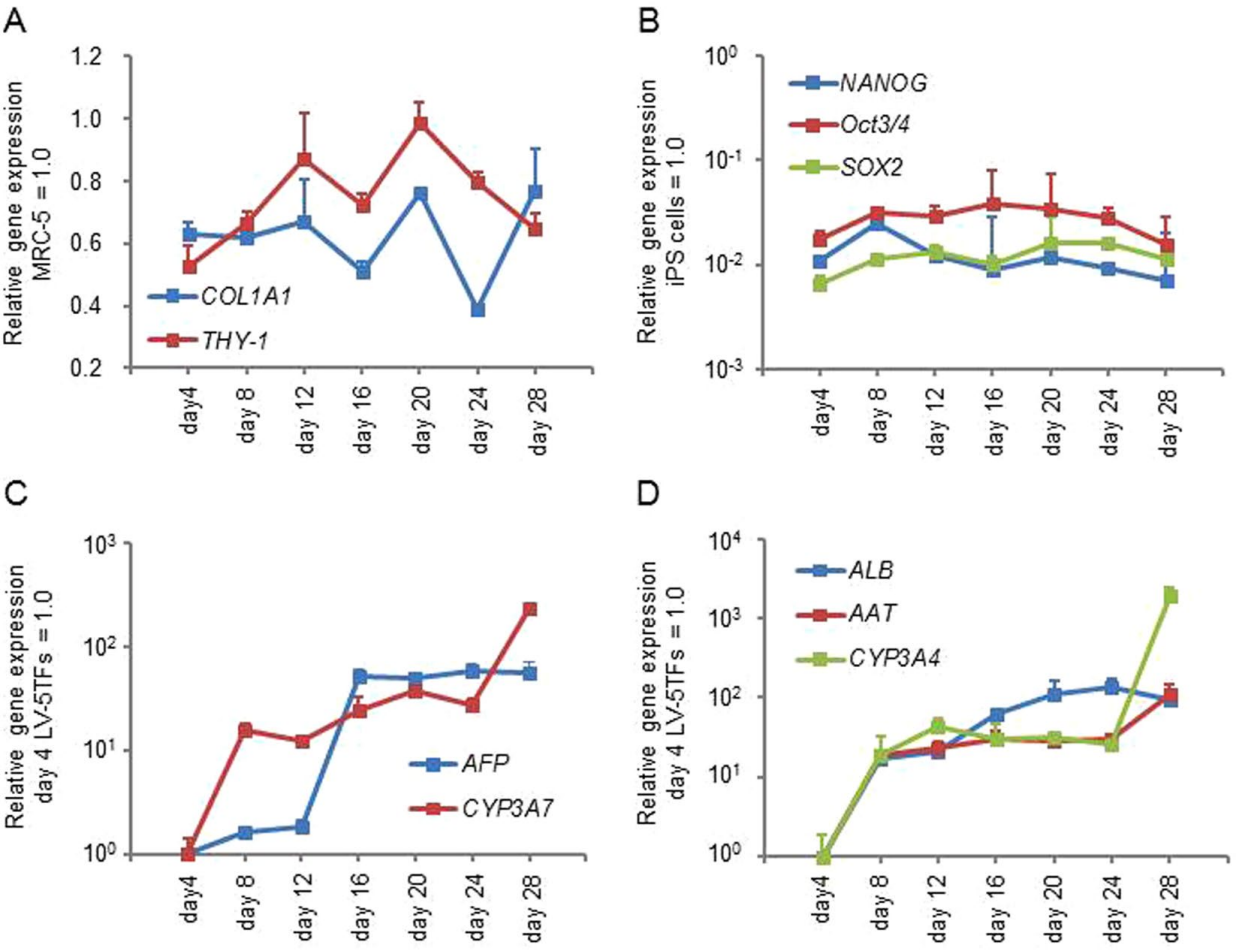
Temporal gene expression profile during the direct reprogramming. MRC5 cells were transduced with LV-5TFs for 12 hr, and cultured until day 28. (**A**) The gene expression levels of fibroblast makers (*COL1A1* and *THY-1*) were measured by real-time RT-PCR. The gene expression levels in MRC5 cells were taken as 1.0. (**B**) The gene expression levels of pluripotent markers (*NANOG*, *Oct3/4*, and *SOX2*) were measured by real-time RT-PCR. The gene expression levels in undifferentiated human iPS cells were taken as 1.0. (**C**) The fetal-specific hepatic gene (*AFP* and *CYP3A7*) expression levels were measured by real-time RT-PCR. The gene expression levels in LV-5TF-transduced MRC5 cells (day 4) were taken as 1.0. (**D**) The matured hepatic gene (*ALB*, *AAT*, and *CYP3A4*) expression levels were measured by real-time RT-PCR. The gene expression levels in LV-5TF-transduced MRC5 cells (day 4) were taken as 1.0. All data are represented as means ± SD (*n* = 3).

Sequential changes in the gene expression levels of fibroblast, pluripotent, fetal hepatic, and adult hepatic markers were evaluated every 4 days after transduction of LV-5TFs. The gene expression levels of fibroblast markers (*COL1A1* and *THY-1*) in hiHeps were similar to those in fibroblasts (Fig. 2A). This result suggests that some fibroblasts retain their status without changing into hiHeps. We confirmed the existence of fibroblast marker-positive cells (13.2%) in hiHeps (Fig. S4). The gene expression levels of pluripotent markers (*NANOG*, *OCT3/4*, and *SOX2*) were significantly lower than those of undifferentiated human iPS cells at any point in the reprogramming process (Fig. 2B). This result suggests that MRC-5 cells were converted into hepatocytes without going through the human iPS cell stage. The gene expression levels of fetal-specific hepatic (*AFP* and *CYP3A7*) (Fig. 2C) and hepatic markers (*ALB*, *AAT*, and *CYP3A4*) (Fig. 2D) increased during the direct reprogramming process. These results suggest that hiHeps retain not only the adult phenotype but also the fetal phenotype.

### Gene expression analysis of transgenes during the direct reprogramming process

Next, we investigated whether the exogenous genes transduced by LV vectors were silenced. Among the 5TFs (ATF5, PROX1, FOXA2, FOXA3, and HNF4A), the expression of exogenous *ATF5*, *PROX1*, *FOXA2*, and *FOXA3* had almost disappeared at day 28 (Fig. S5A). Total gene expression levels (total of the exogenous and endogenous gene expression levels) of *ATF5*, *PROX1*, *FOXA2*, *FOXA3*, and *HNF4A* were also analyzed. The total gene expression levels of *ATF5*, *PROX1*, *FOXA2*, and *FOXA3* in hiHeps (day 28) were still higher than those in the control fibroblasts (day 0) (Fig. S5B). These results suggest that the endogenous *ATF5*, *PROX1*, *FOXA2*, and *FOXA3* were expressed at high levels. On the other hand, exogenous *HNF4A* expression remained at day 28. However, the exogenous *HNF4A* expression level in hiHeps (day 28) was less than 0.01% of the total *HNF4A* expression level (Fig. S5A,B).

### Comparison of hepatic functions between hiHeps and existing hepatocyte models

**Figure 3:**
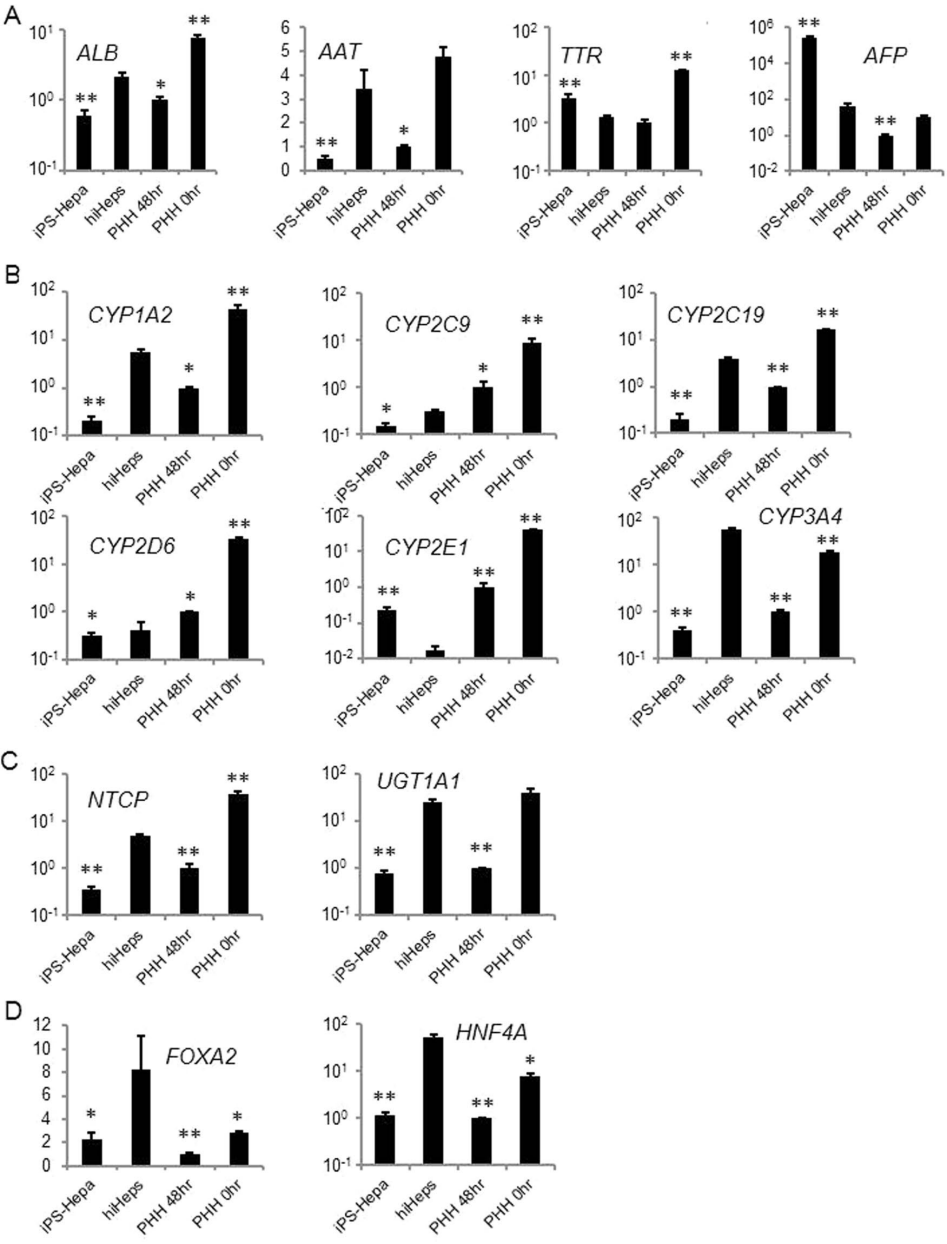
The gene expression profiles of hepatocyte-specific genes and drug metabolic-associated genes. MRC5 cells were transduced with LV-5TFs for 12 hr, and cultured until day 28. (**A**) The hepatic gene (*ALB* and *AAT*) and fetal-specific hepatic gene (*TTR* and *AFP*) expression levels were measured by real-time RT-PCR. The gene expression levels in PHH 48 hr were taken as 1.0. *p < 0.05; **p < 0.01 (vs hiHeps). (**B**) The CYP enzyme gene (*CYP1A2*, *CYP2C9*, *CYP2C19*, *CYP2D6*, *CYP2E1*, and *CYP3A4*) expression levels were measured by real-time RT-PCR. The gene expression levels in PHH 48 hr were taken as 1.0. *p < 0.05; **p < 0.01 (vs hiHeps). (**C**) The gene expression levels of *Na*+*-taurocholate cotransporting polypeptide* (*NTCP*) and *uridine diphosphate glucuronosyltransferase* 1A1 (*UGT1A1*) were measured by real-time RT-PCR. The gene expression levels in PHH 48 hr were taken as 1.0. *p < 0.05; **p < 0.01 (vs hiHeps). (**D**) The hepatic transcription factor (*FOXA2* and *HNF4A*) expression levels were measured by real-time RT-PCR. The gene expression levels in PHH 48 hr were taken as 1.0. *p < 0.05; **p < 0.01 (vs hiHeps). All data are represented as means ± SD (*n* = 3). PHH 48 hr: PHH cultured for 48 hr after plating; PHH 0 hr: PHH collected immediately after thawing.

The hepatic gene expression levels of hiHeps were compared with those of human iPS-Hepa and PHH (Fig. 3). The gene expression levels of *ALB* and *AAT* in hiHeps were higher than those in PHH 48 hr and human iPS-Hepa (Fig. 3A). The gene expression level of fetal hepatic markers (*AFP* and *transthyretin* (*TTR*)) in hiHeps was significantly lower than that in human iPS-Hepa (Fig. 3A). These results suggest that hiHeps have characteristics closer to adult hepatocytes than to human iPS-Hepa. The gene expression levels of *CYP1A2*, *CYP2C19*, and *CYP3A4* in hiHeps were higher than those in PHH 48 hr and human iPS-Hepa (Fig. 3B). On the other hand, the gene expression levels of *CYP2C9* and *CYP2D6* in hiHeps were slightly lower than those in PHH 48 hr (Fig. 3B). The gene expression levels of CYP2E1 in hiHeps were lower than those in human iPS-hepa and PHH 48 hr (Fig. 3B). Moreover, the gene expression levels of the hepatic transporters, *UDP glucuronosyltransferase 1A1* (*UGT1A1*) and *Na* ^+^ *-taurocholate cotransporting polypeptide* (*NTCP*), in hiHeps were higher than those in PHH 48 hr and human iPS-Hepa (Fig. 3C). The gene expression levels of hepatic transcription factors, *FOXA2* and *HNF4A*, in hiHeps were higher than those in PHH 48 hr, human iPS-Hepa, and cryopreserved human hepatocytes (PHH 0 hr) (Fig. 3D). These results suggest that the expression levels of several hepatic markers in hiHeps are higher than those in human iPS-Hepa and PHH 48 hr. However, the hepatic gene expression levels in hiHeps were still lower than those in PHH 0 hr.

**Figure 4:**
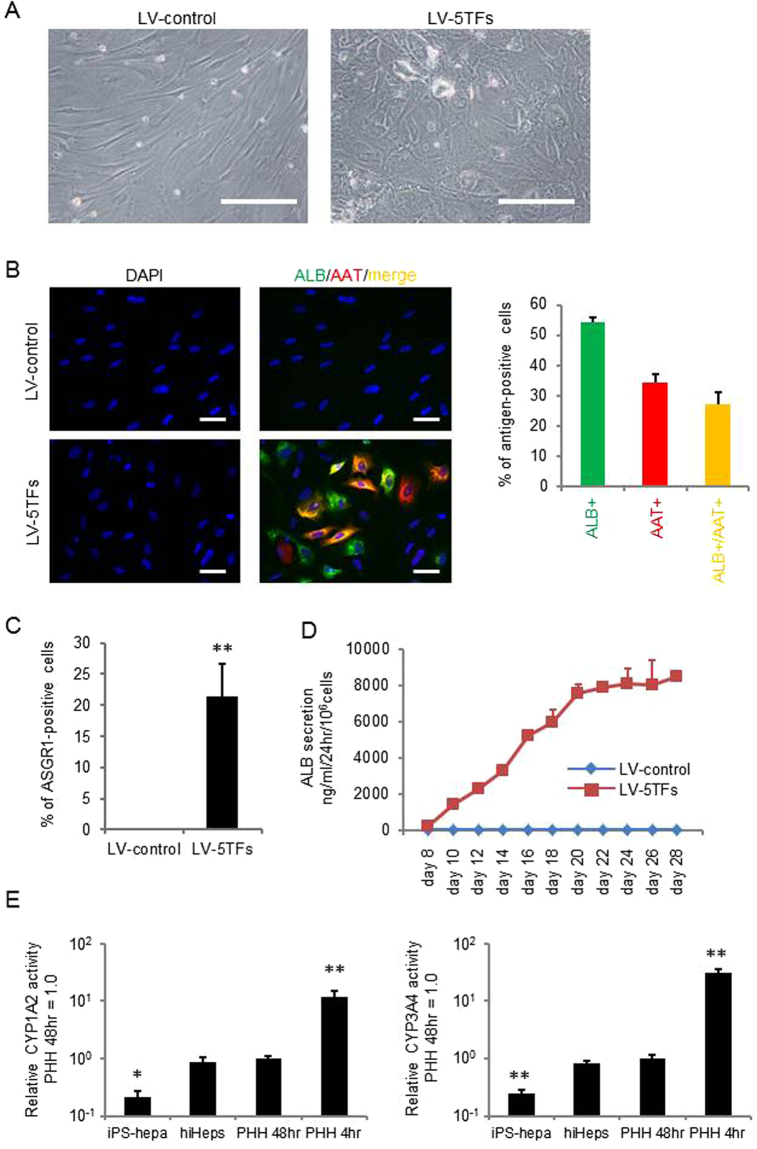
Hepatocyte functionalities of hiHeps. (**A**) MRC5 cells were transduced with LV-5TFs or LV-control for 12 hr, and cultured until day 28. The hiHeps showed hepatic morphology. The scale bars represent 200 μm. (**B**) MRC5 cells were transduced with LV-5TFs or LV-control for 12 hr, and cultured until day 28. These cells were subjected to immunostaining with anti-ALB (green) and anti-AAT (red) antibodies. Nuclei were counterstained with DAPI (blue). The scale bars represent 20 μm. (**C**) The percentage of ASGR1-positive cells in LV-control- or LV-5TFs-transduced cells was examined by FACS. (**D**) The temporal ALB secretion capacity was examined by ELISA in MRC5 cells transduced with LV-5TFs. (**E**) The CYP1A2 and CYP3A4 activities were examined in MRC5 cells transduced with LV-5TFs, human iPS-Hepa, PHH 48 hr, and PHH 4 hr. The CYP1A2 and CYP3A4 activity levels in PHH 48 hr were taken as 1.0. *p < 0.05; **p < 0.01 (vs hiHeps). All data are represented as means ± SD (*n* = 3). PHH 48 hr: PHH cultured for 48 hr after plating; PHH 4 hr: PHH cultured for 4 hr after plating.

Figure 4 provides an evaluation of the hepatic functions of hiHeps. The phase contrast image of hiHeps is shown in Fig. 4A. The expression levels of ALB and AAT in hiHeps were evaluated by immunochemical staining. At day 28, hiHeps were positive for ALB and AAT (Fig. 4B). The percentage of both ALB- and AAT-positive cells was approximately 27%. We also confirmed that the percentage of ASGR1-positive cells in hiHeps was approximately 22% (Fig. 4C). In addition, when we compared the percentage of ASGR1-positve cells between our protocol and Huang’s protocol (Fig. S6), we found that the ASGR1-positive cells were generated more efficiently by our protocol. Next, the ALB secretion levels of hiHeps were examined by ELISA. After day 20, the ALB secretion level of hiHeps was approximately 8,000 ng/ml/24 hr/10^6^ cells (Fig. 4D). The CYP1A2 and CYP3A4 activities in hiHeps were higher than those in human iPS-Hepa, and were similar to those in PHH 48 hr (Fig. 4E). However, CYP1A2 and CYP3A4 activities in hiHeps were still lower than those in PHH cultured for 4  hr (PHH 4 hr). Taken together, these results suggested that hiHeps have a level of hepatic functionality as high as that of PHH 48 hr.

## Discussion

In this study, we demonstrated that highly functional hepatocyte-like cells can be efficiently generated by transducing ATF5, PROX1, FOXA2, FOXA3, and HNF4A into fetal human fibroblasts. Among five the genes, HNF4A plays the most important role, based on the finding that it had the most potent conversion-promoting effect (Figs 1, S2). In agreement with this result, previous reports have used HNF4A for human direct hepatic reprogramming^6,7^. In addition, it is known that HNF4A can control the chromatin structure of target genes^20^, and a majority of the genes expressed in hepatocytes are directly or indirectly regulated by HNF4A^21^. Therefore, it is suggested that HNF4A might provide an appropriate chromatin structure to the other four hepatic transcription factors. From these results, it is conceivable that HNF4A plays a crucial role in direct hepatic reprogramming. The hepatic functionality of hiHeps might be enhanced by modulating the period of expression and the amount of HNF4A.

In this study, we have developed an efficient method for generating functional hiHeps from human fetal fibroblasts. Because stem cells might be contaminated in human fetal fibroblasts, we will need to confirm our findings by using human adult fibroblasts in the future. However, the contribution of stem-cell contamination to direct hepatic reprogramming would be minimum, because the population of stem cells is minor at day 0 and ALB-positive cells accounted for the majority of cells at day 28. Drug metabolism experiments and hepatotoxicity evaluation tests must be also performed to examine whether hiHeps can actually be used for drug-discovery research. In addition, in order to evaluate the possibility of the use of hiHeps for regenerative medicine, we would like to examine whether the transplantation of these cells has a therapeutic effect by using mice with liver injury. Finally, to accelerate these applications, it will be important to construct polycistronic vectors to improve the transduction efficiency and adopt integration-free vectors. Although there are still many problems to be solved, we believe that direct hepatic reprogramming technology will contribute to drug-discovery research and medical development.

## Materials and Methods

### LV (lentiviral) vectors

LV vectors were prepared by using the RIKEN BRC protocol (http://cfm.brc.riken.jp/). The hepatic transcription factors, ATF5, CEBPA, PROX1, FOXA2, FOXA3, HNF1A, HNF4A, HNF6, and GATA4, were amplified by PCR using the primers. The primer sequences used in this study are described in Table S1. Each hepatic transcription factor (TF) was inserted into pCS2-EF-MCS (provided by RIKEN BRC; catalog number: RDB04378), which contains the human elongation factor-1α (EF-1α) promoter, resulting in pCS2-EF-TF. The LV vectors were generated by the transient transfection of four plasmids, pCS2-EF-TF, the packaging construct (pCAG-HIVgp), the VSV-G- and Rev- expressing construct (pCMV-VSV-G-RSV-Rev), and the self-inactivating (SIN) LV vector, into 293T cells. After the LV concentration by ultracentrifugation, the LV copy number (vector particle: VP) was determined by using a Lenti-X real-time RT-PCR Titration Kit (Clontech).

### Direct reprogramming

Human fetal lung fibroblasts of cell line MRC-5 (CCL-171; ATCC) were transduced with a total 125,000 VP/cell of ATF5, PROX1, FOXA2, FOXA3, and HNF4A-expressing lentivirus vectors at 24 hr after seeding. Two days after the transduction, the MRC-5 culture medium (Dulbecco’s modified medium (Wako)) supplemented with 10% FBS and 4 mM L-glutamine was replaced with hepatocyte culture medium (HCM: without epidermal growth factor (EGF); Lonza). At day 28, hiHeps were characterized by real-time RT-PCR analysis, immunochemistry, ELISA, flow cytometry analysis, and CYP activity analysis.

### Facs

Single-cell suspensions of the hiHeps and MRC-5 cells were fixed with 4% paraformaldehyde (PFA) at 4 °C for 10 min, and then incubated with the anti-ASGR1 antibodies (sc-393849, Santa Cruz Biotechnology), followed by the Goat anti-Mouse IgG (H+L) Cross-Adsorbed, Alexa Fluor® 488 antibodies (Thermo Fisher Scientific). Flow cytometry analysis was performed using a FACS LSR Fortessa flow cytometer (BD Biosciences).

### Real-time RT-PCR

Total RNA was isolated from the cells using ISOGENE (NIPPON GENE). cDNA was synthesized using 500 ng of total RNA with a Superscript VILO cDNA synthesis kit (Thermo Fisher Scientific). Real-time RT-PCR was performed with SYBR Green PCR Master Mix (Applied Biosystems) using a StepOnePlus real-time PCR system (Applied Biosystems). ∆∆Ct method was applied for relative quantifications. Each value was normalized against the input determined for the housekeeping gene, *glyceraldehyde 3-phosphate dehydrogenase* (*GAPDH*). The primer sequences used for real-time RT-PCR are described in Table S2.

### Immunohistochemistry

To perform the immunohistochemistry, the cells were fixed with 4% PFA in PBS for 20 min. After incubation with 0.1% Triton X-100 (Sigma) in PBS for 10 min, the cells were blocked with PBS containing 2% FBS and 2% bovine serum albumin (BSA) for 50 min. The cells were incubated with a primary antibody at 4 °C overnight, and then incubated with a secondary antibody at room temperature for 1 hr. All the antibodies are listed in Table S3.

### Elisa

The culture supernatants, which were incubated for 48 hr after fresh medium was added, were collected and analyzed to determine the amount of ALB secretion by ELISA. ELISA kits for ALB were purchased from Bethyl Laboratories. ELISA was performed according to the manufacturer’s instructions. The amount of ALB secretion was calculated according to each standard. The amount of ALB secretion was normalized with cell number.

### Assay for CYP activity

To measure the CYP1A2 and CYP3A4 activity of the cells, we performed lytic assays by using P450-GloTM CYP1A2 and CYP3A4 Assay Kits (Promega). Luciferin-1A2 and luciferin-IPA were used for CYP1A2 and CYP3A4 substrates, respectively. We measured the fluorescence activity with a luminometer (Lumat LB 9507, Berthold) according to the manufacturer’s instructions. The CYP1A2 and CYP3A4 activities were normalized with the protein content per well by using Pierce BCA Protein Assay Kit (Thermo Fisher Scientific) according to the manufacturer’s instructions. Note that the CYP1A2 assay kit mainly detects CYP1A2, but it also detects other CYPs, such as CYP1A1, 1B1, 2A6, 2B6, and 2E1.

### Human iPS cells

Human iPS cells (OHO-iPS cells)^18^ generated from the primary human hepatocytes were maintained on a feeder layer of mitomycin C-treated mouse embryonic fibroblasts (Millipore) with ReproStem (ReproCELL) medium supplemented with 10 ng/ml fibroblast growth factor (FGF) 2 (KATAYAMA Kogyo Kagaku).

### Hepatic differentiation

Before the initiation of hepatic differentiation, human iPS cells were dissociated into clumps by using dispase (Roche Diagnostics) and plated onto BD Matrigel Basement Membrane Matrix Growth Factor Reduced (Becton, Dickinson and Company). These cells were cultured in the mouse embryo fibroblasts-conditioned medium (CM) for 2 days. The differentiation protocol for the induction of definitive endoderm cells, hepatoblast-like cells, and HLCs was based on our previous reports with some modifications^18^. Briefly, in the definitive endoderm differentiation, human iPS cells were cultured with the WNT3A-expressing L cell (CRL2647; ATCC)-conditioned RPMI1640 medium (Sigma) containing 100 ng/mL Activin A (R&D Systems), 1% GlutaMAX (Thermo Fisher Scientific), 0.2% fetal bovine serum (FBS), and 1 × B27 Supplement Minus Vitamin A (Thermo Fisher Scientific) for 4 days. For the induction of hepatoblast-like cells, the definitive endoderm cells were cultured with RPMI1640 medium containing 20 ng/mL bone morphogenetic protein 4 (BMP4) (R&D Systems) and 20 ng/mL FGF4 (R&D Systems), 1% GlutaMAX, and 1 × B27 Supplement Minus Vitamin A for 5 days. To perform the hepatocyte differentiation, the hepatoblasts were cultured with RPMI1640 medium containing 20 ng/mL hepatocyte growth factor (HGF) (R&D Systems), 1% GlutaMAX, and 1 × B27 Supplement Minus Vitamin A for 5 days. Finally, the cells were cultured with the hepatic maturation medium (hepatic maturation medium consists of Hepatocyte Culture Medium (HCM; Lonza, without epidermal growth factor (EGF)) containing 20 ng/mL oncostatin M (OsM) and 3% GlutaMAX) for 11 days.

### Primary human hepatocytes (PHH)

PHH were purchased from VERITAS (lot: OHO). The vials of PHH were rapidly thawed in a shaking water bath at 37 °C, and then the contents of the vial were emptied into prewarmed Cryopreserved Hepatocyte Recovery Medium (CHRM, Thermo Fisher Scientific) and the suspension was centrifuged at 750 rpm for 10 min at room temperature. PHH were seeded at 1.25 × 10^5^ cells/cm^2^ in HCM containing 10% FBS (Thermo Fisher Scientific) onto Cellmatrix Type I-A acid-soluble type I collagen (Nitta Gelatin)-coated plates. The hepatocytes, which were collected immediately after thawing, were named as PHH 0 hr. The hepatocytes, which were cultured 4 hr or 48 hr after plating the cells, were named as PHH 4 hr or PHH 48 hr, respectively.

### Statistic analysis

Statistical analysis was performed using the unpaired two-tailed Student’s *t*-test. All data are represented as means ± SD.

## Electronic supplementary material

supplemental information
