# Supplementary material for: Direct conversion of human fibroblasts into hepatocyte-like cells by ATF5, PROX1, FOXA2, FOXA3, and HNF4A transduction
Source: Sci Rep. 2017 Nov 30;7:16675. doi: 10.1038/s41598-017-16856-7 (PMC5709502; doi:10.1038/s41598-017-16856-7)
Supplement: Supplementary file 1 — supplemental information [file 41598_2017_16856_MOESM1_ESM.pdf]

Title

Direct conversion of human fibroblasts into hepatocyte-like cells by ATF5, PROX1, FOXA2, FOXA3, and HNF4A transduction

Authors

Daiki Nakamori, Hiroki Akamine, Kazuo Takayama,

Fuminori Sakurai, Hiroyuki Mizuguchi

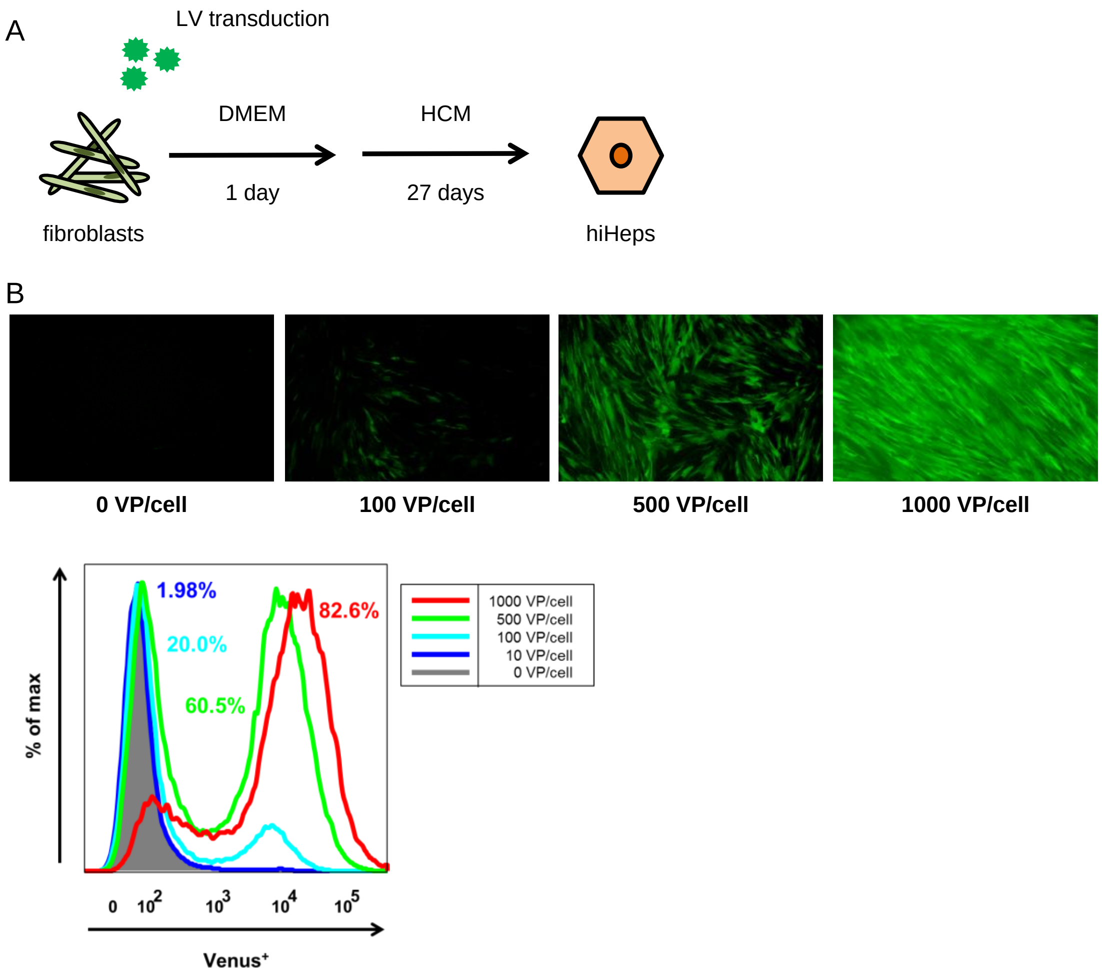

Figure S1 Induction of hepatocyte-like cells from human fetal fibroblasts

(**A**) The procedure for conversion of human fetal fibroblasts (MRC5) cells into hiHeps is presented schematically. MRC5 cells were transduced with hepatic transcription factor-expressing lentivirus vectors (LV-TFs) for 12 hr, and cultured until day 28. (**B**) MRC5 cells were transduced with 0, 100, 500, or 1,000 VP/cell of LV-Venus (modified green fluorescent protein) for 12 hr, and cultured until day 3. The percentage of Venus (green)-positive cells was examined by FACS.

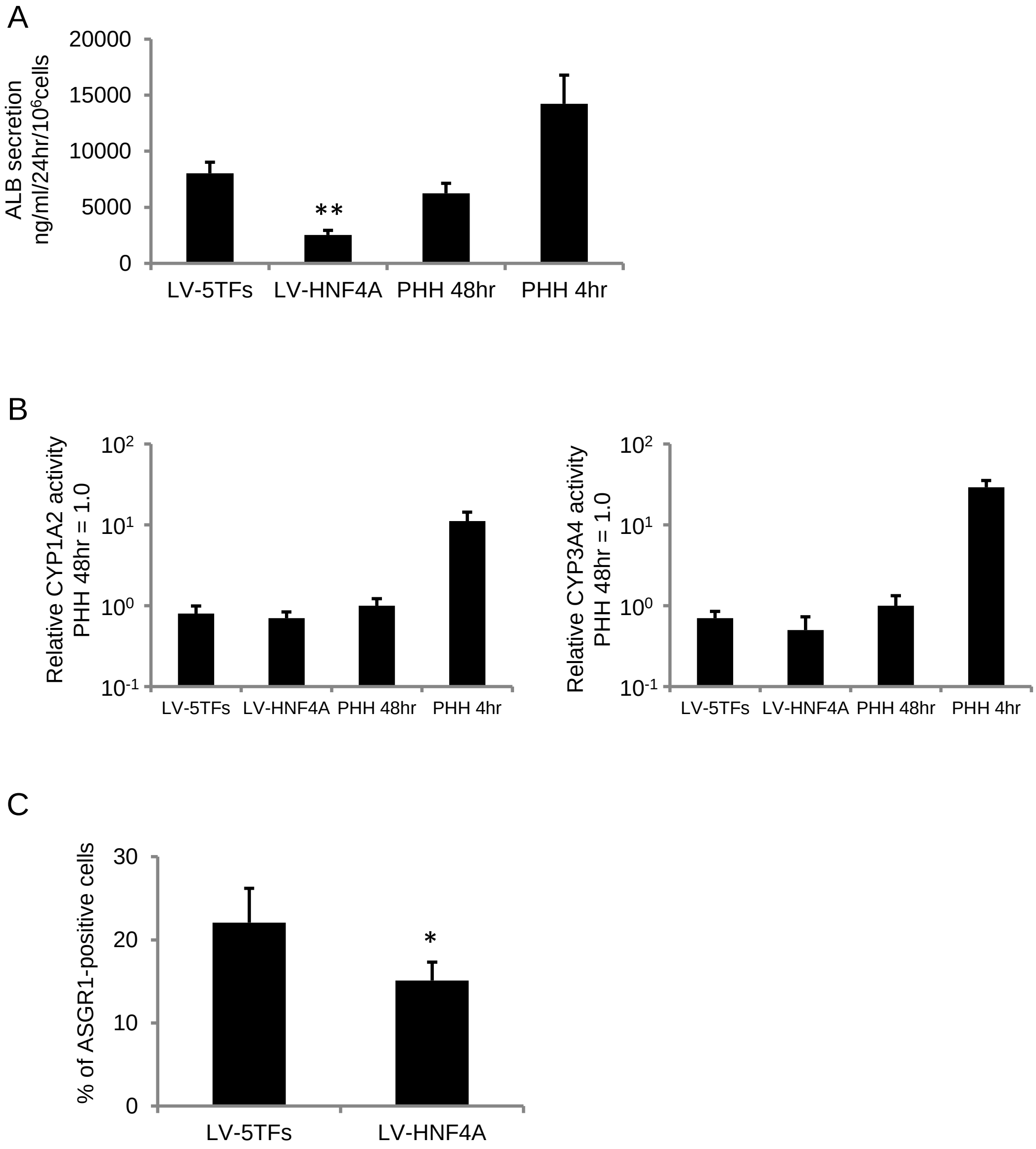

Figure S2 Generation of hiHeps by HNF4A transduction

MRC5 cells were transduced with LV-5TFs or LV-HNF4A for 12 hr, and cultured until day 28. (**A**) The ALB secretion capacity in LV-5TFs- and LV-HNF4A-transduced cells, PHH 48hr, and PHH 4hr was examined by ELISA. $^{**}p < 0.01$ (vs LV-5TFs). (**B**) The CYP1A2 and CYP3A4 activities were examined in LV-5TFs- and LV-HNF4A-transduced cells, PHH 48hr, and PHH 4hr. The CYP1A2 and CYP3A4 activity levels in PHH 48hr were taken as 1.0. (**C**) The percentage of ASGR1-positive cells in LV-5TFs- or LV-HNF4A-transduced cells was examined by FACS. $^{*}p < 0.05$ (vs LV-5TFs). All data are represented as means ± SD (n=3).

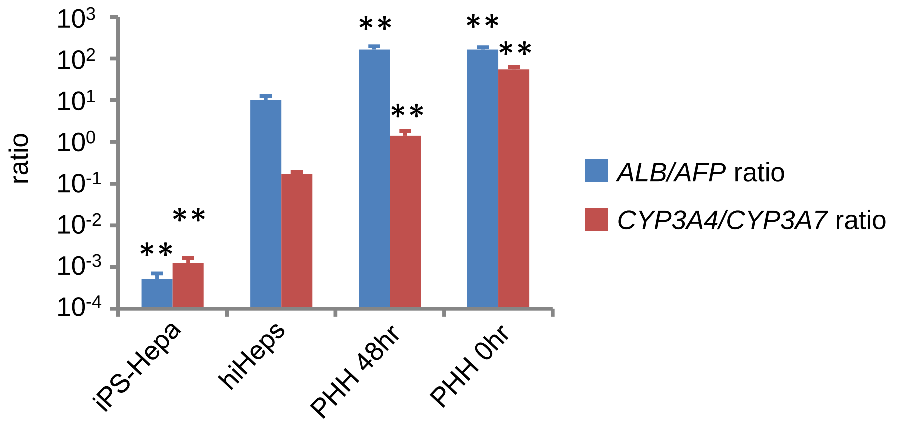

Figure S3 The expression ratios of *ALB/AFP* and *CYP3A4/CYP3A7*

MRC5 cells were transduced with LV-5TFs for 12 hr, and cultured until day 28. The hepatic gene (*ALB* and *CYP3A4*) and fetal-specific hepatic gene (*AFP* and *CYP3A7*) expression levels in iPS-Hepa, hiHeps, PHH 48hr, and PHH 0hr were then measured by real-time RT-PCR. The *ALB/AFP* and *CYP3A4/CYP3A7* ratios were calculated. $^{**}p <$ 0.01 (vs hiHeps).

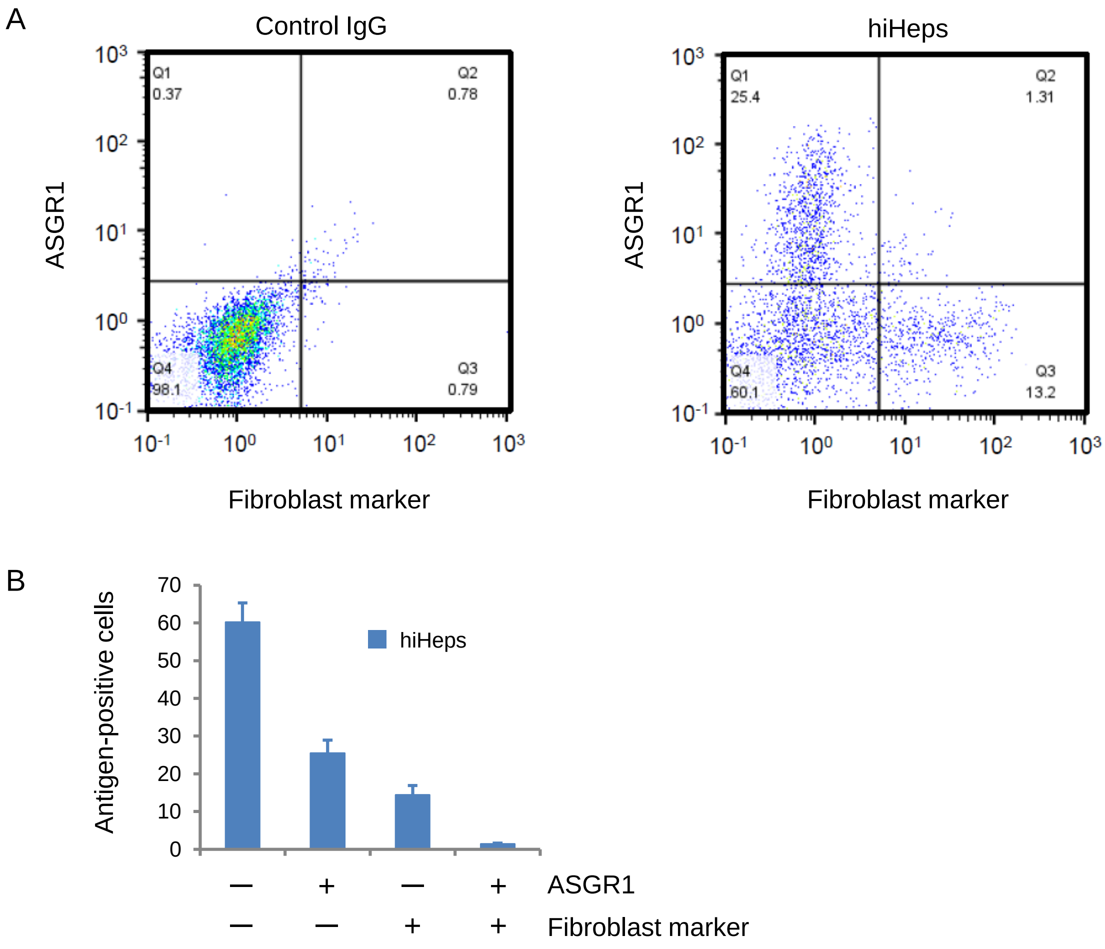

Figure S4　Immunostaining analysis of fibroblast and hepatic markers

MRC5 cells were transduced with LV-5TFs or LV-control for 12 hr, and cultured until day 28. (**A, B**) The percentages of fibroblast marker (THY-1)- and ASGR1-positive cells were examined by FACS analysis. Representative 2D plot data are shown in **figure S4A**. Quantitative data of FACS analysis are shown in **figure S4B**. All data are represented as means ± SD (*n*=3).

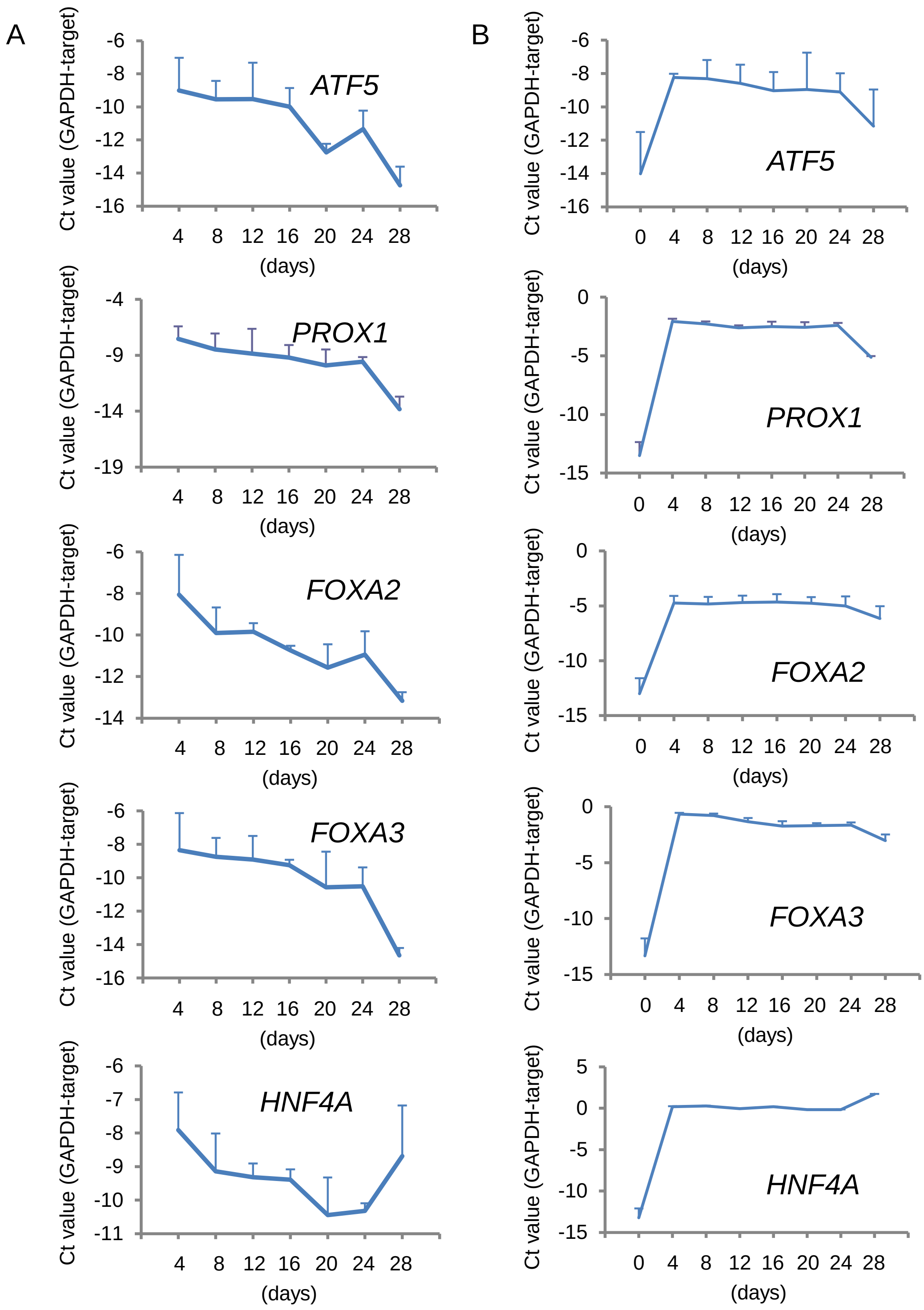

Figure S5 Exogenous and total *ATF5, PROX1, FOXA2, FOXA3,* and *HNF4A* expression profiles during the hepatic reprogramming

MRC5 cells were transduced with LV-5TFs for 12 hr, and cultured until day 28. (**A**) The exogenous gene (*ATF5*, *PROX1*, *FOXA2*, *FOXA3*, and *HNF4A*) expression levels were

measured by qRT-PCR. The gene expression levels in LV-5TF-transduced cells (day 4) were taken as 1.0. (**B**) The total (endogenous and exogenous) gene (*ATF5*, *PROX1*, *FOXA2*, *FOXA3*, and *HNF4A*) expression levels were measured by qRT-PCR. The gene expression levels in LV-5TF-transduced cells (day 0) were taken as 1.0. All data are represented as means ± SD (*n*=3).

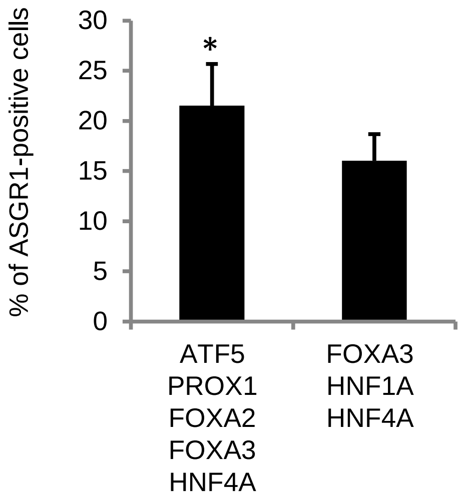

Figure S6 Comparison of hepatic direct reprogramming protocols

The hiHeps were generated by transducing MRC-5 cells with LV-5TFs (ATF5, PROX1, FOXA2, FOXA3, and HNF4A). The hiHeps were also generated by transducing MRC-5 cells with LV-3TFs (FOXA3, HNF1A, and HNF4A). The percentage of ASGR1-positive cells in hiHeps was examined by FACS. *$p < 0.05$ (vs LV-3TFs). All data are represented as means ± SD (n=3).

Table S1   Primers for cloning

Gene Symbol	Genebank Number	Primers (forward/reverse; 5' to 3')
ATF5	NM_ 001193646	TGCTCGAGCCACCATGTCACTCCTGGCGACCCTG
		AAGCGGCCGCCTAGCAGCTACGGGTCCTCTG
PROX1	NM_ 001270616	AACGAATTCGTGATGCCTGACCATGACAGCA
		TGTGCGGCCGCCTACTCATGAAGCAGCTCTTGTAGGCA
CEBPA	NM_004364	TCTCTCGAGCCCATGGAGTCGGCCGACTT
		AGCGGATCCTCACGCGCAGTTGCCCAT
FOXA2	NM_153675	GCGGAATTCAGTATGCTGGGAGCGGTGAA
		GTCGGATCCTTAAGAGGAGTTCATAATGGGCCGGGA
FOXA3	NM_004497	TAACTCGAGGGGATGCTGGGCTCAGTGAA
		CCCGCGGCCGCCTAGGATGCATTAAGCAAAGAGCGGG
HNF1A	NM_000545	GGCGAATTCGCCATGGTTTCTAAACTGAGCCAG
		AGGGCGGCCGCTTACTGGGAGGAAGAGGCCATCT
HNF4A	NM_178849	GAGGAATTCAGAATGCGACTCTCCAAAACCCTC
		CCAGCGGCCGCCTAGATAACTTCCTGCTTGGTGATGGT
HNF6	NM_004498	GCCCTCGAGACGATGAACGCGCAGCTGACCAT
		GTTGCGGCCGCTCATGCTTTGGTACAAGTGCTTGATGAAGAAG
GATA4	NM_002052	CTCCTCGAGACCATGTATCAGAGCTTGGCCATGG
		GAGGAATTCTTACGCAGTGATTATGTCCCCGTGAC

Table S2 Primers for real-time RT-PCR analysis

Gene Symbol	Primers (forward/reverse; 5' to 3')
ALB	GCACAGAATCCTTGGTGAACAG/ATGGAAGGTGAATGTTTTCAGCA
AAT	ACTGTCAACTTCGGGGACAC/CATGCCTAAACGCTTCATCA
CYP3A4	AAGTCGCCTCGAAGATACACA/AAGGAGAGAACACTGCTCGTG
COL1A1	GAGGGCCAAGACGAAGACATC/CAGATCACGTCATCGCACAAC
THY-1	CTAGTGGACCAGAGCCTTCG/TGGAGTGCACACGTGTAGGT
NANOG	AGAAGGCCTCAGCACCTAC/GGCCTGATTGTTCCAGGATT
Oct3/4	CTTGAATCCCGAATGGAAAGGG/GTGTATATCCCAGGGTGATCCTC
SOX2	GGCAGCTACAGCATGATGATGCAGGAGC/CTGGTCATGGAGTTGTACTGCAGG
AFP	TGGGACCCGAACTTTCCA/GGCCACATCCAGGACTAGTTTC
CYP3A7	AAGGTCGCCTCAAAGAGACA/TGCACTTTCTGCTGGACATC
ATF5	GACCGCAAGCAAAAGAAGAG/GGCCTTGTAAACCTCGATGA
PROX1	TTGACATTGGAGTGAAAAGGACG/TGCTCAGAACCTTGGGGATTC
CEBPA	CCAAGAAGTCGGTGGACAAG/AGGCGGTCATTGTCACTGGT
FOXA2	GCGACCCCAAGACCTACAG/GGTTCTGCCGGTAGAAGGG
FOXA3	TCATGTAGGAGTTGAGGGGG/GAAGATGGAGGCCCATGAC
HNF1A	TACACCACTCTGGCAGCCACACT/CGGTGGGTACATTGGTGACAGAAC
HNF4A	CGTCATCGTTGCCAACACAAT/GGGCCACTCACACATCTGTC
HNF6	AACCCTGGAGCAAACTCAAA/AAAAAGCCCAGGTTGGTCTT
GATA4	CATCAAGACGGAGCCTGGCC/TGACTGTCGGCCAAGACCAG
TTR	TCATCGTCTGCTCCTCCTCT/AGGTGTCATCAGCAGCCTTT
CYP1A2	CAATCAGGTGGTGGTGTCAG/GCTCCTGGACTGTTTTCTGC
CYP2C9	GGACAGAGACGACAAGCACA/CATCTGTGTAGGGCATGTGG
CYP2C19	ACTTGGAGCTGGGACAGAGA/CATCTGTGTAGGGCATGTGG
CYP2D6	CTTTCGCCCCAACGGTCTC/TTTTGGAAGCGTAGGACCTTG
CYP2E1	ACCCGAGACACCATTTTCAG/TCCAGCACACACTCGTTTTC
NTCP	ATCTTGGTCTGTGGCTGCTC/AGAAGGTGGAGCAGGTGGT
UGT1A1	TAAGTGGCTACCCCAAAACG/GCTTTGCATTGTCCATCTGA
GAPDH	GGTGGTCTCCTCTGACTTCAACA/GTGGTCGTTGAGGGCAATG

Table S3 Antibodies for immunohistochemistry

Antigen	Type	Company
ALB	goat	Bethyl Laboratories
AAT	rabbit	Dako
Alexa Fluor 488 anti-goat IgG	donkey	Thermo Fisher Scientific
Alexa Fluor 594 anti-rabbit IgG	donkey	Thermo Fisher Scientific
